# Association Between Low-Level Lead Exposure and Serum Gamma-Glutamyl Transferase Concentrations as a Biomarker of Oxidative Stress in U.S. Adolescents Aged 12–19 Years

**DOI:** 10.3390/ijerph23010028

**Published:** 2025-12-24

**Authors:** Wenping Hu, Tanya T. LeBlanc, Audrey F. Pennington, Cheryl R. Cornwell, Paul B. Allwood

**Affiliations:** National Center for Environmental Health, Centers for Disease Control and Prevention, Atlanta, GA 30341, USA

**Keywords:** blood lead level, gamma-glutamyl transferase, oxidative stress, adolescent, NHANES

## Abstract

Objectives: This study aims to investigate the potential relationship between low-level lead exposure and serum gamma-glutamyl transferase (GGT) concentrations, which may serve as a biomarker of oxidative stress in U.S. adolescents. Methods: We used NHANES data from 1999 to 2000 to 2017–2018. Analyses were limited to adolescents aged 12–19 years with blood lead levels (BLLs) below 5 µg/dL (*n* = 11,978). BLLs were categorized into either two groups based on the median BLL (i.e., <0.70 µg/dL and ≥0.70 µg/dL) or four quartiles (i.e., quartile 1: <0.46 µg/dL; quartile 2: 0.46–<0.70 µg/dL; quartile 3: 0.70–<1.00 µg/dL; and quartile 4: ≥1.00 µg/dL). Multivariable linear regression analysis was conducted to assess the association between BLLs and serum GGT concentrations among U.S. adolescents aged 12–19 years. Results: Adolescents with BLLs ≥ 0.70 µg/dL showed significantly higher serum GGT concentrations compared to those with BLLs < 0.70 µg/dL (geometric means: 13.94 vs. 12.80 U/L; *p* < 0.001). The multivariable linear regression analysis indicated that, after adjusting for potential confounding factors, natural log-transformed serum GGT concentrations were higher among adolescents in BLL quartile 4 compared to those in BLL quartile 1 (β-coefficient = 0.0607; 95% CI: 0.0306, 0.0908). However, no significant associations were observed between BLLs and serum GGT concentrations among adolescents in BLL quartiles 2 and 3 when compared to quartile 1. Conclusions: In the present study, we observed that higher BLLs were associated with higher serum GGT concentrations in U.S. adolescents aged 12–19 years. Further research is needed to substantiate the positive relationship between BLLs and serum GGT and explore the mechanisms underlying their interaction with oxidative stress in adolescents.

## 1. Introduction

Lead is a toxic heavy metal that occurs naturally in the environment. Its widespread industrial and domestic use has caused extensive contamination, ongoing human exposure, and significant public health concerns [1,2]. Over the past several decades, efforts to control lead sources, such as removing lead from gasoline and paint, have resulted in a substantial decline in population-level lead exposure [3,4]. Nevertheless, lead cannot be eliminated from the environment; low-level human exposure to lead persists. The low-level lead exposure has been linked to adverse health effects on neurodevelopment and cognition [5,6]. It is necessary to continually investigate the effects of low-level lead exposure (<5.0 µg/dL), as a better understanding of those effects can inform public health interventions designed to reduce lead exposure.

Gamma-glutamyl transferase (GGT) is an enzyme found throughout the human body [7,8]. It plays an essential role in regulating the metabolism of glutathione, an abundant intracellular antioxidant. GGT maintains redox homeostasis by hydrolyzing extracellular glutathione and providing recovered cysteine for de novo intracellular glutathione synthesis [8]. Research indicates that serum GGT may serve as a biomarker for oxidative stress [9].

Oxidative stress, which may be induced by lead exposure, is considered as one of the important mechanisms underlying lead toxicity [5,10,11]. There is growing interest in investigating the relationship between blood lead levels (BLLs) and serum GGT concentrations as a biomarker of oxidative stress. Previous studies have found that BLLs are positively associated with serum GGT concentrations in adults [12,13,14]. However, Al-Neamy et al., reported inconsistent findings, showing no significant difference in serum GGT concentrations between industrial workers with higher BLLs and non-industrial workers with lower BLLs [15]. These discrepancies may arise from variations in age groups studied, levels of lead exposure, duration of exposure, or differences between occupationally exposed and general populations [12,13,14,15]. Although the relationship between lead exposure and oxidative stress has been explored in adults and occupationally exposed groups, there have been few studies examining the relationship between BLLs, particularly those that are considered low, and serum GGT in adolescents. Given the distinct physiological and developmental characteristics of adolescents, as well as their vulnerability to lead exposure, the objective of the present study is to investigate this potential relationship among U.S. adolescents aged 12–19 years using a large, nationally representative dataset from the National Health and Nutrition Examination Survey (NHANES) across multiple survey cycles from 1999 to 2000 to 2017–2018.

## 2. Methods

### 2.1. Study Population

The National Health and Nutrition Examination Survey, conducted by the National Center for Health Statistics at the Centers for Disease Control and Prevention, is a cross-sectional, nationally representative survey of the non-institutionalized civilian population of the United States. Comprehensive information about NHANES can be found online at https://www.cdc.gov/nchs/nhanes (accessed on 16 July 2025). For this study, data from ten consecutive cycles of NHANES (i.e., 1999–2000, 2001–2002, 2003–2004, 2005–2006, 2007–2008, 2009–2010, 2011–2012, 2013–2014, 2015–2016, and 2017–2018) were combined. A total of 13,715 adolescents aged 12–19 years were selected after excluding those with missing values for sample weight (*n* = 551), BLL (*n* = 3234), and serum GGT concentration (*n* = 2332). Additionally, we excluded 43 adolescents who had BLLs ≥ 5.0 µg/dL. Of the remaining participants, 1594 adolescents with missing values on covariates of interest were also excluded. Ultimately, 11,978 adolescents were included in the analysis. 

### 2.2. Measurement of Blood Lead and Serum GGT

Whole blood samples were obtained through venipuncture from eligible adolescents during health examinations. The collected specimens were initially kept at refrigerated temperatures (2–8 °C) and then transferred to pre-screened plastic cryovials for freezing at ≤−70 °C until testing. BLLs were measured using graphite furnace atomic absorption spectrometry from 1999 to 2000 to 2001–2002 [16] and inductively coupled plasma mass spectrometry from 2003 to 2004 to 2017–2018 [16,17]. The limit of detection for blood lead varied by survey cycle: 0.30 µg/dL from 1999 to 2000 to 2001–2002, 0.25–0.28 µg/dL in 2003–2004, 0.25 µg/dL from 2005 to 2006 to 2011–2012, and 0.07 µg/dL from 2013 to 2014 to 2017–2018. For adolescents with BLLs below the lower limit of detection, their BLLs were replaced with values equal to the detection limit divided by the square root of 2 [16].

Serum GGT analyses were conducted using an enzymatic rate method. The following analyzers were employed during different periods: Hitachi Model 704 multichannel analyzer (Boehringer Mannheim Diagnostics, Indianapolis, IN, USA) from 1999–2000 to 2001–2002; Beckman Synchron LX20 (Beckman Coulter Diagnostics, Brea, CA, USA), Beckman UniCel^®^ DxC 800 Synchron Clinical System (Beckman Coulter Diagnostics, Brea, CA, USA), or Beckman UniCel^®^ DxC 660i Synchron Clinical System (Beckman Coulter Diagnostics, Brea, CA, USA) from 2003 to 2004 to 2015–2016); and Roche Cobas 6000 Chemistry Analyzer in 2017–2018 (Roche Diagnostics, Indianapolis, IN, USA) [16]. In NHANES 2017–2018, a Log Deming regression was used to adjust serum GGT concentrations due to changes in laboratory equipment and analytical methods [16].

### 2.3. Covariates

Several variables were selected for analysis, including age (12–15 years and 16–19 years), sex (male and female), race/Hispanic origin (Hispanic, non-Hispanic White, non-Hispanic Black, and Other), education level of household reference person, ratio of family income to poverty (PIR), serum cotinine concentration, and body mass index (BMI). The education level of the household reference person was divided into three categories: less than high school, high school, and some college or above. PIR is the ratio of family income to poverty as defined by the U.S. Department of Health and Human Services. It was categorized into three levels: <1, 1–2, and ≥2. A PIR < 1 indicates that family income is below the poverty threshold. Serum cotinine concentrations were categorized into four quartiles: quartile 1: <0.017 ng/mL; quartile 2: 0.017–<0.059 ng/mL; quartile 3: 0.059–0.755 ng/mL; and quartile 4: ≥0.755 ng/mL. BMI is a measure of weight relative to height and is calculated as measured body weight (in kilograms) divided by the square of measured height (in meters) in NHANES. Based on sex-specific BMI-for-age percentiles for children and teens aged 2 through 19 years [18], BMI values were categorized as follows: less than the 5th percentile (underweight), from the 5th to less than the 85th percentile (normal weight), from the 85th to less than the 95th percentile (overweight), at or above the 95th percentile (obesity).

### 2.4. Statistical Analyses

All statistical analyses were conducted using the Survey Sampling and Analysis Procedures in SAS (version 9.4, SAS Institute Inc., Cary, NC, USA) to account for the complex survey design and weights in NHANES. The appropriate sample weights across the combined ten survey cycles of NHANES (1999–2018) were calculated according to NHANES guidance. We calculated the weighted percentages of adolescent characteristics. Geometric means (GM) and standard errors for BLLs and serum GGT concentrations were also calculated overall and by selected characteristics. Moreover, we estimated the GM of serum GGT concentrations in two BLL groups categorized based on the median BLLs (<0.70 µg/dL vs. ≥0.70 µg/dL). Such estimates enable a clear comparison between lower and higher lead exposure groups.

We conducted multivariable linear regression analyses to assess the potential relationships between BLLs (predictor) and serum GGT concentrations (outcome). Two models were used: (I) a crude model, not adjusted for covariates, and (II) an adjusted model, adjusted for age, sex, race/Hispanic origin, education level of household reference person, PIR, serum cotinine concentration, and BMI. In both the overall model and subgroup analyses, serum GGT concentrations and age of adolescents were considered continuous variables. BLLs were categorized into four quartiles: quartile 1: <0.46 µg/dL; quartile 2: 0.46–<0.70 µg/dL; quartile 3: 0.70–1.00 µg/dL; and quartile 4: ≥1.00 µg/dL; moreover, due to the non-normal distribution of serum GGT concentrations, natural log-transformed values were used in our multivariable linear regression analysis. Subgroup analyses were conducted using a stratified multivariable regression model by specifying each subgroup (i.e., age and sex) in the DOMAIN statement. Statistical significance was defined at a *p*-value of <0.05.

## 3. Results

The BLLs and serum GGT concentrations, categorized by the characteristics of the study population, are presented in Table 1. The weighted study population consisted of 51.3% males and 48.7% females, with 51.3% of the adolescents aged 12–15 years and 48.7% aged 16–19 years. Regarding race/Hispanic origin, the adolescents identified as follows: Hispanic (18.9%), non-Hispanic White (60.0%), non-Hispanic Black (13.7%), and other (7.4%) (Table 1). The overall GM of BLLs and serum GGT concentrations were 0.70 µg/dL and 13.36 U/L, respectively. Moreover, the GM of serum GGT concentrations was higher in male adolescents compared to female adolescents (15.04 U/L vs. 11.79 U/L). The GM of serum GGT concentrations increased with age, increasing from 12.52 U/L in adolescents aged 12–15 years to 14.31 U/L in adolescents aged 16–19 years (Table 1).

Table 2 presents the GM of serum concentrations based on BLLs categorized as <0.70 µg/dL versus ≥0.70 µg/dL. Overall, the adolescents with BLLs ≥ 0.70 µg/dL showed significantly higher serum GGT concentrations compared to those with BLLs < 0.70 µg/dL (13.94 U/L vs. 12.80 U/L; *p* < 0.001). When analyzing subgroups by age, adolescents aged 12–15 years with BLLs ≥ 0.70 µg/dL had significantly higher serum GGT concentrations than adolescents aged 12–15 years with BLLs < 0.70 µg/dL (12.96 U/L vs. 12.07 U/L; *p* < 0.001). Similarly, among adolescents aged 16–19 years, those with BLLs ≥ 0.70 µg/dL also showed significantly higher serum GGT concentrations compared to those with BLLs < 0.70 µg/dL (15.11 U/L vs. 13.58 U/L; *p* < 0.001). When analyzing subgroups by sex, significantly higher serum GGT concentration was observed in female adolescents with BLLs ≥ 0.70 µg/dL when compared to female adolescents with BLLs < 0.70 µg/dL (12.06 U/L vs. 11.62 U/L; *p* = 0.016); but this was not observed in male adolescents (*p* = 0.062) (Table 2).

**Figure 1:**
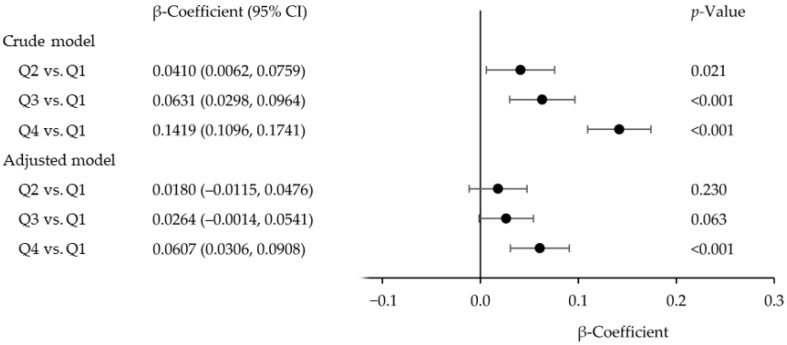
Regression β-coefficients (95% confidence interval [CI]) for the association between blood lead levels (Q1: quartile 1, <0.46 µg/dL; Q2: quartile 2, 0.46–<0.70 µg/dL; Q3: quartile 3, 0.70–1.00 µg/dL; Q4: quartile 4, ≥1.00 µg/dL) and serum gamma-glutamyl transferase concentrations in adolescents aged 12–19 years. NHANES 1999–2000 to 2017–2018.

The relationship between BLLs and serum GGT concentrations in our analysis is shown in Figure 1. In the adjusted model, the β-coefficient for adolescents in BLL quartile 4, relative to adolescents in BLL quartile 1 (reference), was 0.0607 (95% confidence interval [CI]: 0.0306, 0.0908; *p* < 0.001). The β-coefficients for adolescents in BLL quartile 2 (0.0180 [95% (CI): −0.0115, 0.0476]; *p* = 0.230) and quartile 3 (0.0264 [95% (CI): −0.0014, 0.0541]; *p* = 0.063) were not statistically significant when compared to those for adolescents in BLL quartile 1. For the crude model, the β-coefficient for adolescents in BLL quartiles 2, 3, and 4 were 0.0410 (95% CI: 0.0062, 0.0759; *p* = 0.021), 0.0631 (95% CI: 0.0298, 0.0964; *p* < 0.001), and 0.1419 (95% CI: 0.1096, 0.1741; *p* < 0.001), respectively, compared to adolescents in BLL quartile 1. Both the adjusted and crude models demonstrated that BLLs in quartile 4 were associated with higher serum GGT concentrations compared to BLLs in quartile 1 (Figure 1).

Through subgroup analysis, we further examined the potential relationship between BLLs and serum GGT concentration by age and sex. In the adjusted model, a statistically significant association between BLLs (quartile 4 vs. quartile 1) and serum GGT concentrations was observed in adolescents aged 12–15 years (β-coefficient = 0.0484, 95% CI: 0.0152, 0.0816; *p* = 0.005), as well as in adolescents aged 16–19 years (β-coefficient = 0.0655, 95% CI: 0.0161, 0.1149; *p* = 0.010). Similarly, the positive association between BLLs (quartile 4 vs. quartile 1) and serum GGT concentrations was statistically significant in male adolescents (β-coefficient = 0.0447, 95% CI: 0.0053, 0.0841; *p* = 0.026), and in female adolescents (β-coefficient = 0.0649, 95% CI: 0.0215, 0.1084; *p* = 0.004) (Table 3).

The subgroup analysis by sex using the median BLL groups (Table 2) showed a statistically significant association between BLLs and serum GGT concentrations in females, but not in males. In contrast, when BLLs were categorized into quartiles (Table 3), the association in the highest quartile (Quartile 4: ≥1.00 µg/dL) was statistically significant for both sexes. This discrepancy suggests that the quartile approach may be more sensitive in detecting associations at higher lead exposure levels than median categorization. Moreover, the association between BLLs and serum GGT concentrations may differ by sex, which could contribute to the variation in statistical significance observed between males and females.

## 4. Discussion

Lead exposure poses health risks to the human body. Research continues to show that lead exposure, even at low levels, is associated with physical and intellectual health problems [19,20]. For instance, Evans et al. [20] found that BLLs below 10 μg/dL were inversely associated with reading and math scores among third-grade children, and that 13% of reading failures and 14.8% of math failures could be attributed to lead exposure with BLLs 5–9 μg/dL in comparison to those with BLLs 0–4 μg/dL. In the present study, we explored the potential relationship between BLLs, specifically at low levels (GM: 0.70 μg/dL; range: 0.05–<5 μg/dL), and serum GGT concentrations. Our results revealed a positive association between low-level lead exposure (BLL quartile 4) and serum GGT concentrations among U.S. adolescents.

Several studies have evaluated serum GGT concentrations in adults suffering from occupational lead exposure [14,21,22]. For instance, Mazumdar and Goswami found that serum GGT concentrations were significantly higher in a lead-exposed group of plastic industry workers with BLLs 59.6 ± 6.5 µg/dL compared to a control group with BLLs 12.3 ± 3.2 µg/dL (63 ± 7 U/L vs. 19 ± 5 U/L; *p* < 0.001) [21]. Lee et al. [14] conducted a study on 1654 steel workers whose BLLs were classified into three BLL tertiles (i.e., tertile 1: GM: 0.82 µg/dL; range: 0.1–1.1 µg/dL; tertile 2: GM: 1.38 µg/dL; range: 1.2–1.6 µg/dL; tertile 3: GM: 2.15 µg/dL; range: 1.7–4.6 µg/dL). The odds ratio for having an elevated serum GGT concentration in BLL tertile 3 compared to BLL tertile 1 (reference) was 2.74 (*p* < 0.001), 1.83 (*p* = 0.013), and 1.81 (*p* = 0.016) in the unadjusted model and two other adjusted models, respectively. With respect to the studies conducted in the general population, Li et al. [23] explored the potential relationship between BLLs and serum GGT concentration in adults using NHANES data from 2011 to 2018 (*n* = 15,328) and found a positive association between log2-transformed BLLs (continuous) and serum GGT concentrations (β-coefficient = 1.79, 95% CI: 0.68, 2.89; *p* < 0.01). Moreover, based on the data from the Korean National Environmental Health Survey (2015–2017), Kim et al. [24] demonstrated that serum GGT concentrations increase with increasing BLLs (*p* for trend < 0.001). The positive association between BLLs and serum GGT concentration in adolescents observed in the present study is consistent with findings from previous studies conducted on adults [13,14,21,22,23,24]. These studies indicate that a positive relationship between BLLs and serum GGT concentrations may exist regardless of whether BLLs and serum GGT concentrations were assessed in lead-exposed occupational samples or in the general population.

The mechanism underlying the positive association between BLLs and serum GGT concentrations is unclear. Lead exposure may induce oxidative stress, which occurs when the generation of reactive oxygen species exceeds the capacity of the antioxidant defense system against those free radicals. The induced oxidative stress plays an important role in the pathophysiology of lead toxicity [25]. Adverse health effects attributable to lead exposure, such as hypertension, kidney dysfunction, and cognitive disorders, have been associated with oxidative stress [26,27]. Glutathione is a tripeptide containing cysteine that has a reactive thiol group (-SH). During the biological process to protect against oxidative stress induced by lead exposure, glutathione interacts directly with free radicals to protect cells from damage; however, this process can lead to depletion of the intracellular glutathione [26,27]. On the other hand, glutathione is the most common substrate for GGT, an enzyme located on the outer membrane of cells. GGT can specifically cleave the gamma-glutamyl bond and degrade extracellular glutathione, allowing the cell to use its amino acid components for the intracellular synthesis of glutathione. Therefore, the synthesized intracellular glutathione contributes to the maintenance of intracellular redox homeostasis [8]. Due to the essential role played by GGT in modulating redox homeostasis within cells as well as in their surroundings, it is not surprising that alterations in its expression and activity have been associated with the pathogenesis of various diseases related to oxidative stress, such as cardiovascular diseases, cancer, and lung inflammation [8,28]. Meanwhile, there is increasing evidence suggesting that serum GGT may be a biomarker for oxidative stress [9,12,29,30,31]. For instance, Lee et al. [12] demonstrated that BLLs were positively associated with serum GGT concentrations, but inversely associated with serum vitamin C, carotenoids, and vitamin E concentrations in U.S. adults aged ≥20 years. In the present study, our findings indicated that the positive association between BLLs and serum GGT concentrations was statistically significant; however, the observed changes in serum GGT related to variations in BLLs were modest and remained within the normal range. Nonetheless, these modest increases in serum GGT may still reflect increased oxidative stress among adolescents exposed to low levels of lead.

The present study is subject to several limitations. First, this cross-sectional study could not assess whether there is a causal-effect relationship between BLLs and serum GGT concentration. Moreover, blood lead and serum GGT levels were measured at one point in time, which might not accurately reflect long-term lead exposure and oxidative stress development. A prospective study with repeated measurements over time might be more appropriate to explore the potential relationship between BLLs and serum GGT concentrations. Second, the multivariable linear regression model was adjusted for some factors (e.g., age), but the observed association may be confounded by unincluded and/or unmeasured factors. For instance, there may be simultaneous exposure to toxic metals other than lead, which might induce oxidative stress related to a change in serum GGT concentrations. Moreover, although serum GGT may serve as a biomarker of oxidative stress, it is a non-specific enzyme that can be elevated due to various underlying metabolic conditions, such as non-alcoholic fatty liver disease. Consequently, elevations in GGT may potentially confound the observed association between BLLs and serum GGT concentrations. Third, there are a variety of oxidative stress biomarkers; the commonly used oxidative stress biomarkers used to verify associations with BLLs were malondialdehyde, glutathione, glutathione peroxidase levels, superoxide dismutase, and catalase activities [32]. Previous epidemiological studies in children and adults from the general population have reported inconsistent findings regarding the association between low-level lead exposure and oxidative stress, as assessed by various biomarkers of oxidative stress [27]. In the present study, we were unable to assess lead-induced oxidative stress using the biomarkers other than serum GGT concentrations, due to the unavailability of those biomarkers in NHANES. Indeed, the inclusion of multiple biomarkers may increase the ability to detect lead-induced oxidative stress and enhance our understanding of the nature of the oxidative stress-related damage that occurs in the body [27].

## 5. Conclusions

The present study demonstrates a significant association between low-level lead exposure and increased serum GGT concentrations in U.S. adolescents, suggesting a potential link between lead exposure and oxidative stress during growth and development. These findings underscore the importance of continued public health efforts to minimize lead exposure in this population. Additional research to explore the mechanism underlying the potential relationship between BLLs and serum GGT concentrations, as well as their interaction with oxidative stress, could clarify the observed association. If the association between low-level lead exposure and oxidative stress is further substantiated, understanding the extent to which low-level lead exposure contributes to oxidative stress, as reflected by serum GGT, could help inform preventive strategies to regulate the antioxidative defense system against lead toxicity.

## Figures and Tables

**Table 1:** Blood lead levels and serum gamma-glutamyl transferase concentrations in adolescents aged 12–19 years stratified by study characteristics. NHANES 1999–2000 to 2017–2018.

Variable	*n*	% (Weighted) ^a^	Lead GM (SE) ^b^, µg/dL	GGT GM (SE) ^b^, U/L
Sex				
Male	6053	51.3	0.83 (0.01)	15.04 (0.11)
Female	5925	48.7	0.58 (0.01)	11.79 (0.10)
Age				
12–15 years	6115	51.3	0.71 (0.01)	12.52 (0.10)
16–19 years	5863	48.7	0.68 (0.01)	14.31 (0.15)
Race/Hispanic origin				
Hispanic	4294	18.9	0.68 (0.02)	13.72 (0.15)
Non-Hispanic White	3369	60.0	0.68 (0.01)	12.78 (0.12)
Non-Hispanic Black	3416	13.7	0.81 (0.02)	15.44 (0.14)
Other	899	7.4	0.72 (0.03)	13.70 (0.28)
Ratio of family income to poverty ^c^				
<1	3766	22.5	0.80 (0.02)	14.06 (0.15)
1–2	3254	22.8	0.74 (0.02)	13.48 (0.15)
≥2	4958	54.7	0.64 (0.01)	13.03 (0.12)
Education level of the household reference person				
Less than high school	3936	20.7	0.80 (0.02)	13.94 (0.16)
High school or some college	6167	56.1	0.70 (0.01)	13.51 (0.10)
College graduate or above	1875	23.3	0.60 (0.01)	12.52 (0.17)
Serum cotinine level (ng/mL)				
Quartile 1 (<0.017)	2405	24.5	0.53 (0.01)	12.39 (0.15)
Quartile 2 (0.017–<0.059)	3144	25.3	0.67 (0.01)	12.86 (0.15)
Quartile 3 (0.059–0.755)	3410	25.2	0.75 (0.01)	13.50 (0.16)
Quartile 4 (≥0.755)	3019	25.0	0.88 (0.02)	14.78 (0.18)
Body mass index				
Underweight	351	3.4	0.75 (0.03)	12.51 (0.39)
Healthy weight	7105	61.5	0.71 (0.01)	12.35 (0.09)
Overweight	1990	16.1	0.69 (0.01)	13.44 (0.19)
Obesity	2532	18.9	0.64 (0.01)	17.46 (0.29)
Total	11,978	100.0	0.70 (0.01)	13.36 (0.09)

^a^ The total may not precisely add up due to rounding. ^b^ GM (SE): geometric mean (standard error). ^c^ The ratio of family income to poverty as defined by the U.S. Department of Health and Human Services. The value of <1 indicates that family income is below the poverty threshold.

**Table 2:** Serum gamma-glutamyl transferase concentrations in adolescents aged 12–19 years, stratified by two blood lead levels. NHANES 1999–2000 to 2017–2018.

	Serum Gamma-Glutamyl Transferase (U/L)				
Variable	BLLs < 0.70 µg/dL ^a^		BLLs ≥ 0.70 µg/dL ^a^		*p*-Value
	Geometric Mean	Standard Error	Geometric Mean	Standard Error	
Overall	12.80	0.13	13.94	0.12	<0.001
Age					
12–15 years	12.07	0.15	12.96	0.11	<0.001
16–19 years	13.58	0.21	15.11	0.18	<0.001
Sex					
Male	14.78	0.18	15.21	0.14	0.062
Female	11.62	0.13	12.06	0.14	0.016

^a^ BLLs: blood lead levels; Median BLLs = 0.70 µg/dL.

**Table 3:** Regression β-coefficient (95% CI) for serum gamma-glutamyl transferase concentrations by quartile of blood lead levels, stratified by age and sex in adolescents aged 12–19 years. NHANES 1999–2000 to 2017–2018 ^a b c^.

		Crude Model		Adjusted Model	
		β-Coefficient (95% CI)	*p*-Value	β-Coefficient (95% CI)	*p*-Value
Age					
12–15 years	Q2 vs. Q1	0.0171 (−0.0234, 0.0575)	0.406	0.0094 (−0.0226, 0.0413)	0.564
	Q3 vs. Q1	0.0425 (0.0048, 0.0801)	0.027	0.0184 (−0.0155, 0.0523)	0.285
	Q4 vs. Q1	0.1086 (0.0735, 0.1436)	<0.001	0.0484 (0.0152, 0.0816)	0.005
16–19 years	Q2 vs. Q1	0.0664 (0.0106, 0.1222)	0.020	0.0224 (−0.0251, 0.0699)	0.353
	Q3 vs. Q1	0.0849 (0.0366, 0.1332)	0.001	0.0314 (−0.0112, 0.0740)	0.147
	Q4 vs. Q1	0.1908 (0.1396, 0.2419)	<0.001	0.0655 (0.0161, 0.1149)	0.010
Sex					
Male	Q2 vs. Q1	−0.0334 (−0.0824, 0.0155)	0.179	−0.0176 (−0.0579, 0.0226)	0.388
	Q3 vs. Q1	−0.0098 (−0.0554, 0.0359)	0.673	0.0090 (−0.0324, 0.0503)	0.669
	Q4 vs. Q1	0.0201 (−0.0212, 0.0613)	0.338	0.0447 (0.0053, 0.0841)	0.026
Female	Q2 vs. Q1	0.0386 (−0.0033, 0.0805)	0.070	0.0377 (−0.0019, 0.0774)	0.062
	Q3 vs. Q1	0.0354 (−0.0053, 0.0760)	0.088	0.0272 (−0.0105, 0.0648)	0.156
	Q4 vs. Q1	0.0817 (0.0388, 0.1245)	<0.001	0.0649 (0.0215, 0.1084)	0.004

^a^ Adjusted model: adjusted for age, gender, race/ethnicity, serum cotinine concentration, ratio of family income to poverty, education level of household reference person, and body mass index. ^b^ Four quartiles (Q1–Q4) of blood lead levels: Q1, <0.46 µg/dL; Q2, 0.46–<0.70 µg/dL; Q3, 0.70–1.00 µg/dL; Q4, ≥1.00 µg/dL. ^c^ 95% CI: 95% confidence interval.

## Data Availability

All data are presented in the article. For further information, contact the corresponding author.

## Abbreviations

The following abbreviations are used in this manuscript:

GGT	Gamma-glutamyl transferase
NHANES	The National Health and Nutrition Examination Survey
BLLs	Blood lead levels
GM	Geometric mean
CDC	Centers for Disease Control and Prevention
PIR	Ratio of family income to poverty
BMI	Body mass index
CI	Confidence interval
SE	Standard error
